# Dr. Kinglake's Correction of a Mis-Statement

**Published:** 1803-12-01

**Authors:** Robert Kinglake

**Affiliations:** Taunton


### *To the Editors of the Medical and Physical Journal.*

GENTLEMEN,

IN the true spirit of just investigation, you have, as liberally as laudably, endeavoured to discourage the communications of anonymous writers, by refusing them the authority of medical evidence; you will therefore readily coincide with me in the propriety of correcting the misrepresentation contained in a paper of your Journal, (No. 50, p. 362) signed "Constant Reader," by publishing the subsequent remarks on that subject, extracted from the last volume of Dr. Duncan's Annals of Medicine. Indeed, it would be compromising a gross mis-statement, to withhold this intelligence from your numerous readers.

The paper alluded to appeared to me, *prima facie*, to be as exceptionable for its want of internal as external evidence. My own words, in reply to the author, bear testimony to this opinion. These were,* "It must be readily perceived, that the communication (in question) breathes a spirit of levity and wrangling, wholly inconsistent with the grave decorum due to the investigation, and decision, of a philosophical subject; nor does the veil of anonymous concealment, which the author has thought fit to assume, impart to the statement the genuine stamp of unquestionable authenticity."

It will be clearly seen how commendably the scientific labours of the Authors of the Annals of Medicine are regulated by a scrupulous attention to the interests of truth and philosophical impartiality, by the following extract, in which they discover a solicitude to rectify a mistake, which, if unexplained, may have operated to the prejudice of a doctrine they profess rather to disapprove than to defend.

*Extract of an article in the last volume of Dr. Duncan's Annals of Medicine, entitled, "Notice of Dr. Kinglake's Proposal of the topical Use of cold Water in the Gout."*

"The following letter, addressed to the Editors of the Medical and Physical Journal, has appeared in the 50th Number of that publication:"

"Gentlemen,

* See Medical and Physical Journal, No. 51, p. 442.

"Gentlemen,

"As Dr. Kinglake calls loudly upon his medical brethren, to communicate any observations which may enable him to make up his mind as soon as possible, the following case, on the authenticity of which he may depend, is much at his service.

"The late celebrated Dr. Gregory, of Edinbugh, father of the present Professor of Medicine in that University, was very liable to gout. A friend of the present writer called on him one evening, and found him bathing his feet in cold water. He observed to the Doctor, that he was doing what he would hardly recommend to his patients. No, said the Doctor, but this application mitigates pain, which I am unwilling to bear, and I have hitherto experienced no bad effects from it. The next morning the Doctor, to the regret of every admirer of science, and of professional liberality, was found lifeless in his bed.

"But why trouble ourselves any farther about the gout? One gentleman can completely pump out the gout from the system in a day or two! and another has discovered a remedy, a few spoons full of which will enable a cripple to rise from his bed, put on a pair of tight shoes, eat a good dinner, and afterwards dance a hornpipe. Surely one remedy for a disease is sufficient; my only fear is, that unless we can find out some new diseases, for which as yet there are no remedies, the Faculty must starve!

"A Constant Reader."

"Although from the conclusion of this letter, it is evident that the writer of it means to treat Dr. Kinglake's proposal with ridicule, yet it will naturally be concluded, that what he has asserted respecting the late Dr. Gregory is strictly true; and, if true, it would certainly have been an important fact.

"It was indeed a very current report at the time of Dr. Gregory's death, that he had been accustomed to bathe his feet in cold water, and had done so the evening before that event took place. But, upon the authority of those who had the best opportunities of knowing, we can inform the public that this report is entirely groundless; that, on the contrary, Dr. Gregory, who had often been subjected to severe attacks of the gout, was at much pains to keep his feet warm; that he had no symptoms of gout for many months before his death, having enjoyed a much longer interval of health than usual; that, on the very day preceding his death, he had dined abroad with some friends,

 and and had supped with his family; that he had not bathed his feet the night before he died, and was left by his son, the present Dr. Gregory, at half an hour after twelve o'clock, preparing his lecture for next day, and apparently in perfect health; but was found lifeless next morning; and that from the undisturbed condition of the bed-clothes it was concluded he had died without a struggle."

The Editors then proceed to say, "But although the sudden death of Dr. Gregory affords no objection against Dr. Kinglake's proposal, yet we are very far from asserting, that the application of cold wet cloths to a part inflamed, and painful from gout, is a safe practice."

Such is the refutation which a zealous and unbiassed love of scientific truth, has enabled me to quote on "A Constant Reader's" unqualified assertion, that his statement of Dr. Gregory's case possessed an "authenticity on which he (Dr. Kinglake) may depend." *Humanum est errare.* I am, &c.

*Taunton, Oct* 28, 1803. ROBERT KINGLAKE.

